# Tailoring ZE21B Alloy with Nature-Inspired Extracellular Matrix Secreted by Micro-Patterned Smooth Muscle Cells and Endothelial Cells to Promote Surface Biocompatibility

**DOI:** 10.3390/ijms23063180

**Published:** 2022-03-16

**Authors:** Changsheng Liu, Lan Chen, Kun Zhang, Jingan Li, Shaokang Guan

**Affiliations:** 1 School of Materials Science and Engineering, Zhengzhou University, 100 Science Road, Zhengzhou 450001, China; lcszzu123@163.com (C.L.); chenlan@zzu.edu.cn (L.C.); skguan@zzu.edu.cn (S.G.); 2 Henan Key Laboratory of Advanced Magnesium Alloy & Key Laboratory of Materials Processing and Mold Technology (Ministry of Education), School of Material Science and Engineering, Zhengzhou University, 100 Science Road, Zhengzhou 450001, China; 3 School of Life Science, Zhengzhou University, 100 Science Road, Zhengzhou 450001, China; zhangkun@zzu.edu.cn

**Keywords:** cardiovascular materials, ZE21B alloy, biocompatibility, dECM coating, hyaluronic acid micro-patterns

## Abstract

Delayed surface endothelialization is a bottleneck that restricts the further application of cardiovascular stents. It has been reported that the nature-inspired extracellular matrix (ECM) secreted by the hyaluronic acid (HA) micro-patterned smooth muscle cells (SMC) and endothelial cells (EC) can significantly promote surface endothelialization. However, this ECM coating obtained by decellularized method (dECM) is difficult to obtain directly on the surface of degradable magnesium (Mg) alloy. In this study, the method of obtaining bionic dECM by micro-patterning SMC/EC was further improved, and the nature-inspired ECM was prepared onto the Mg-Zn-Y-Nd (ZE21B) alloy surface by self-assembly. The results showed that the ECM coating not only improved surface endothelialization of ZE21B alloy, but also presented better blood compatibility, anti-hyperplasia, and anti-inflammation functions. The innovation and significance of the study is to overcome the disadvantage of traditional dECM coating and further expand the application of dECM coating to the surface of degradable materials and materials with different shapes.

## 1. Introduction

Cardiovascular disease is the main cause of premature death worldwide; it not only brings great suffering to human beings, but also brings many countries great economic losses [1]. At present, the main method for treating coronary artery diseases is stent implantation; however, during implantation the endothelial cell (EC) layer will be destroyed, which will cause inflammation and excessive smooth muscle cell (SMC) proliferation. More severely, it will cause intravascular restenosis and endanger patients’ life [2,3]. The reason can be summarized as the insufficient endothelialization, anti-proliferation, anti-coagulation, and anti-inflammatory function of the vascular stent materials [4]. Extracellular matrix (ECM) is a complex network, which is composed of collagen, fibronectin, elastin, proteoglycans, glycosaminoglycans, and many types of factors (such as vascular endothelial growth factor, thrombomodulin, tissue pathway factor inhibitor, etc.) [5]. ECM can also mediate the characteristics of biophysical and biochemical signals, leading to the dynamic response of cells to the surrounding microenvironment, and can transmit signals between ECM and cells to maintain the balance of all aspects in the body, including effectively improving cell living environment, provide support for cell adhesion and migration, and regulate cell life cycle [6,7]. Therefore, using the structural and functional properties of ECM and depositing it on the surface of vascular stent materials is recognized as an effective surface modification method [8,9,10].

Tu et al. have prepared the EC-ECM and SMC-ECM, respectively, by decellularized method on the materials surface, and discovered the SMC-ECM could promote EC growth, while the EC-ECM could enhance nitric oxide (NO) release from the adherent EC and inhibit platelet adhesion [11,12]. However, the EC and SMC cultured in vitro are different from the cells in vivo, thus their ECM are also different. Our research found that the hyaluronic acid (HA) micro-pattern was able to control the EC and SMC to the physiological phenotypes [13,14]. The further study also proved the ECM secreted by HA patterned EC demonstrated better biocompatibility than the ECM from non-patterned EC [15,16]. In the recent work, the SMC-ECM and EC-ECM secreted from the HA patterned SMC and EC were combined by alternative culture and acellularization of cells, presenting stronger biocompatibility [17]. Nevertheless, this novel decellularized method has its limitations on application for biodegradable materials and materials with different shapes.

In this study, we obtain the nature-inspired ECM via the previous decellularized method [17] and dispersed it into normal saline (NS) by ultrasonic concussion. Subsequently, the dispersed ECM was conjugated on to the biodegradable Mg-Zn-Y-Nd (ZE21B) alloy by self-assembly technology. This novel method was anticipated to overcome the application disadvantage of the ECM prepared by decellularized method (dECM) on the biodegradable materials and materials of different shapes, such as biodegradable magnesium (Mg) alloy stents.

## 2. Results and Discussion

### 2.1. Preparation and Characterization of Biomimetic Vascular Basement Membrane ECM Coating

**Figure 1:**
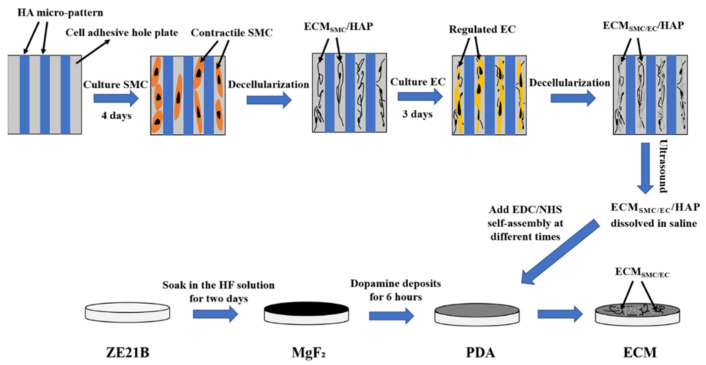
Preparation processes of nature-inspired ECM coating onto ZE21B by self-assembly.

We improved the core method of Han et al. [17]: firstly, a 25 μm wide hyaluronic acid (HA, Bloomage Biotechnology Co., Ltd., Jinan, China) pattern was imprinted on the 6-adhesive well plate to regulate the SMC to contractile phenotype, and next the contractile SMC were decellularized after they were cultured for four days to obtain the dECM of SMC; secondly, the EC were seeded on the well plate that were coated with HA patterned SMC-dECM, and decellularized after cultured for three days to obtain the dECM of EC. Here, a double-layered ECM composed of the SMC-dECM and EC-dECM was obtained (labeled as ECM_SMC/EC_/HAP); the further process was adding NS into each well, placing the plate in an ultrasonic water bath for 20 min to dissolve the ECM_SMC/EC_/HAP dispersed into NS. The ZE21B alloy prepared in our laboratory was cut into small discs with their thickness of 3 mm and a diameter of 10 mm. After grinding and polishing [18], the ZE21B samples were immersed in a hydrogen fluoride (HF) solution for two days to prepare the passivation layer (the treated samples were labeled as MgF_2_) [19], and then the MgF_2_ samples were immersed in the dopamine solution for 6 h to obtain the poly-dopamine intermediate layer (the samples were labeled as PDA) to covalently connect the ECM with sample by the EDC/NHS activation method [20]. The self-assembly of ECM_SMC/EC_/HAP on PDA samples took 6 h, 12 h, and 24 h, respectively (the corresponding samples were marked as ECM 6 h, ECM 12 h, ECM 24 h, respectively). The preparation process of the ECM coating on the ZE21B was shown in Figure 1.

**Figure 2:**
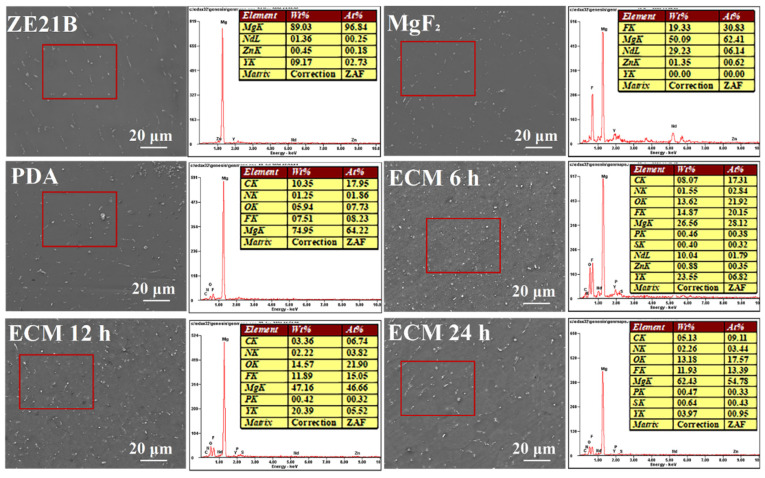
SEM images and EDS spectra of ZE21B, MgF_2_, PDA, ECM 6 h, ECM 12 h, and ECM 24 h samples.

The scanning electron microscopy (SEM) image of each sample is shown in Figure 2. All the samples presented smooth morphologies, with several white dots scattered on the surfaces, which may be the precipitated grains of the Mg alloy. Here, the ECM 6 h, ECM 12 h, and ECM 24 h samples seemed to possessed more white dots when compared with the ZE21B, MgF_2_, and PDA samples, which could be attributed to the complexation between self-assembled ECM and degradation products of Mg alloy. The EDS results further verified the ECM self-assembly on the MgF_2_ and PDA coated ZE21B alloy: The bare ZE21B showed its high Mg element of 89.03%, while after the MgF_2_ layer preparation, the Mg element decreased to 50.09%. However, the F element appeared and increased to nearly 20%. After the preparation of PDA film, its characteristic element, N, increased to 1.25%. There were a large number of protein and amino acid components in the extracellular matrix, and the N element was a main component. With the extension of the self-assembly time, the N element sequentially increased from 1.55% to 2.22% and to 2.26% on the ECM samples, due to the amino acid components in the ECM, suggesting successful conjugation of ECM on the Mg alloy.

**Figure 3:**
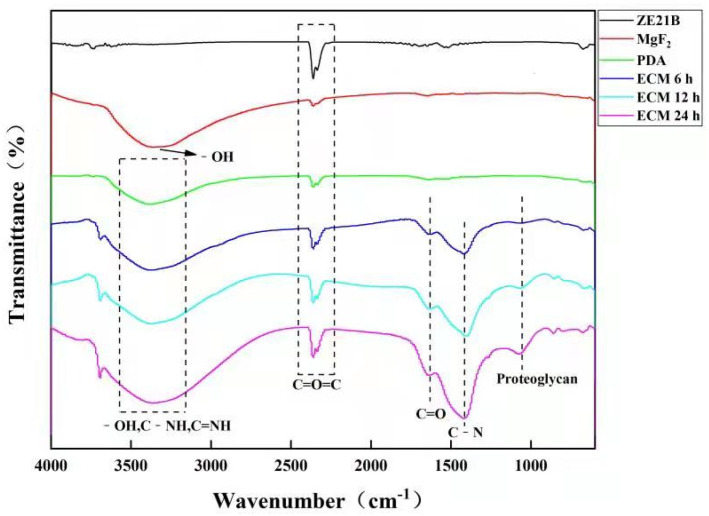
FTIR spectra of ZE21B, MgF_2_, PDA ECM 6 h, ECM 12 h, and ECM 24 h samples.

The characteristic functional group changes of different samples are shown in Figure 3. Compared with the ZE21B sample, other samples displayed obvious new peaks on their curves. The broad peaks in the range of 3500–3100 cm^−1^ were considered as -OH, C-NH, and C=NH. The peak at about 1640 cm^−1^ corresponded to the C=O stretching in the ECM. Compared with the MgF_2_ and PDA samples, the ECM sample showed a new peak at 1400 cm^−1^, which was considered as the C-N bending vibration of the amide III band. The peak near 1080 cm^−1^ was also considered to be the proteoglycan peak in ECM [21].

**Figure 4:**
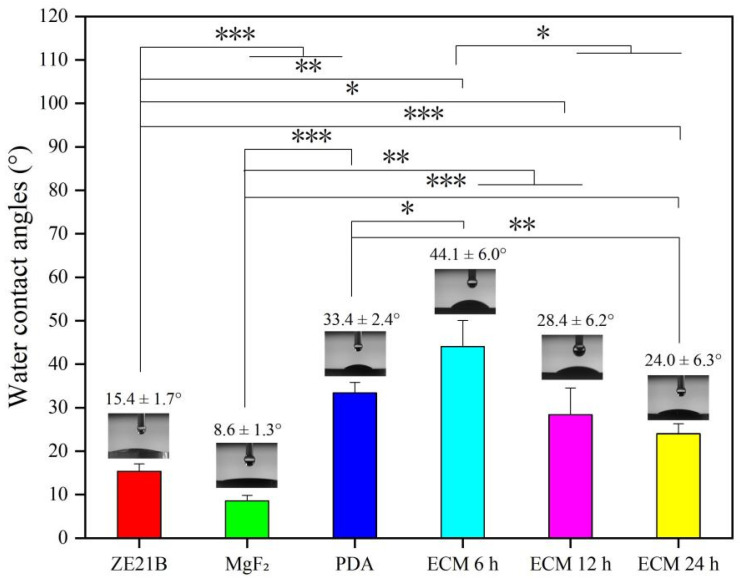
Water contact angles’ analysis of ZE21B, MgF_2_, PDA, ECM 6 h, ECM 12 h, and ECM 24 h samples (mean ± SD, *n* = 3, * *p* < 0.05, ** *p* < 0.01, and *** *p* < 0.001).

**Figure 5:**
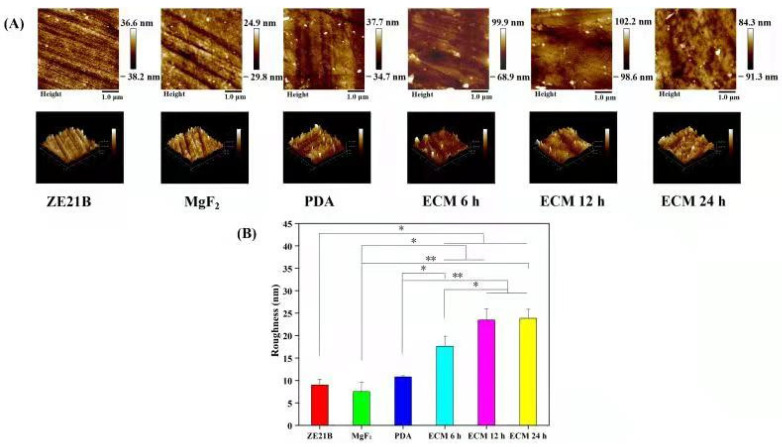
(**A**) 2D and 3D AFM images of ZE21B, MgF_2_, PDA, ECM 6 h, ECM 12 h, and ECM 24 h samples. (**B**) The average surface roughness (Rq) of ZE21B, MgF2, PDA, ECM 6 h, ECM 12 h, and ECM 24 h samples (mean ± SD, *n* = 3, * *p* < 0.05, and ** *p* < 0.01).

Water contact angles (WCA) less than 90° are usually considered as a hydrophilic surface [22]. The hydrophilic surface can adsorb more proteins and growth factors, which helps cell adhesion and diffusion [22,23]. Figure 4 shows that the WCA of pure ZE21B alloy was 15.9 ± 1.7°, and the values further decreased to 8.6 ± 1.3° after MgF_2_ preparation due to the dense MgF_2_ coating, while after PDA film deposition it increased to 33.4 ± 2.4°, which may be caused by the exposure of the benzene ring and other hydrophobic groups, including -OH and NH_2_, etc.. The WCA increased to 44.1 ± 6.9° after self-assembly of ECM 6 h, which may be due to the uneven distribution of the ECM within 6 h. With the self-assembly time increased, the ECM distribution was more and more uniform, therefore, the WCA decreased again and maintained the stable values at 12 h and 24 h. The AFM results showed that ECM samples presented rougher surfaces compared to the other samples (Figure 5), which indirectly proved that the ECM had been self-assembled to the ZE21B surface.

**Figure 6:**
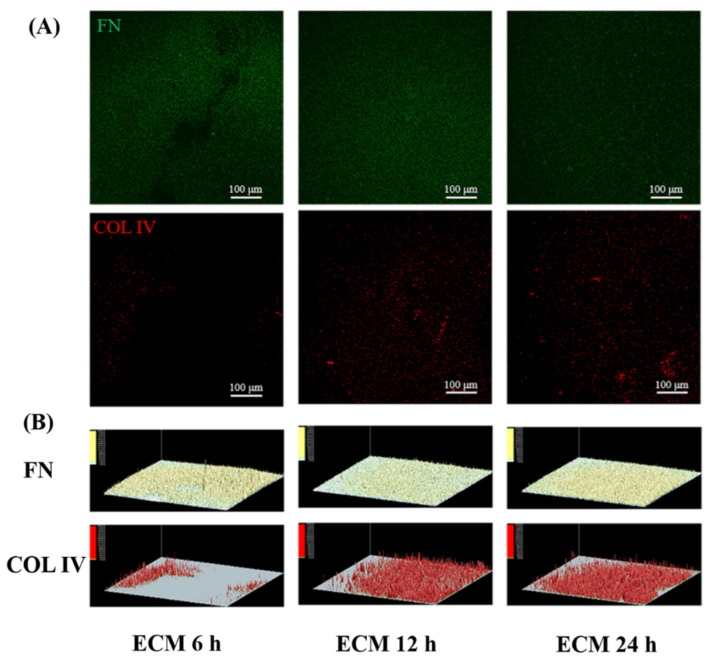
(**A**) The fluorescence images of FN (green color) and COL IV (red color) distributed on the ECM 6 h, ECM 12 h, and ECM 24 h samples; (**B**) 3D images of FN and COL IV fluorescence intensity generated by Ipwin32 software.

Figure 6 showed that the FN and COL IV on the ECM 24 h sample seemed more uniform compared to these two proteins on the ECM 12 h and ECM 6 h samples, which indicated that prolonging the grafting time can make the ECM more evenly distributed on the surface. These results suggested that the grafting rates of the FN and COL IV were different with the extension of grafting time, which may lead to different FN/COL IV ratio and distribution of the three ECM coatings. It is known that ECM is composed of more complex biomolecules, while the proportion and construction of these biomolecules in the ECM secreted by natural cells are more precise and orderly, which is quite different from the ECM secreted by cells cultured in vitro and are far from being completely simulated by the current artificial construction methods. FN and COL IV are only some representative molecules in ECM components, and their proportion varies with the change of grafting time. Therefore, it can be inferred that other components of ECM are affected by grafting time.

### 2.2. Electrochemical Test

**Figure 7:**
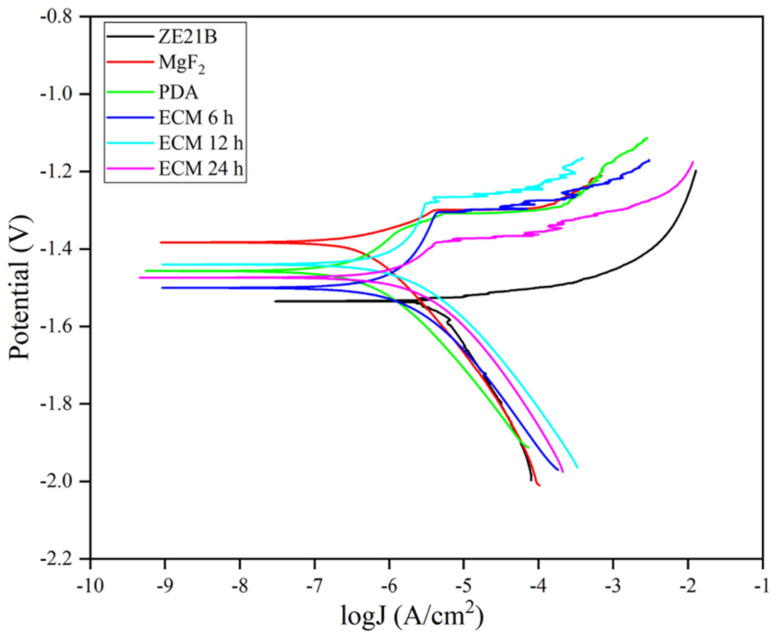
Tafel curve images of ZE21B, MgF_2_, PDA, ECM 6 h, ECM 12 h, and ECM 24 h samples.

The corrosion performance of each sample was evaluated by electrochemical testing. The self-corrosion potential (Ecorr) generally reflects the ease with which the sample begins to corrode, and the corrosion current density (Icorr) can reflect the rate of corrosion of the sample. Figure 7 and Table 1 showed that ZE21B alloy possessed the smallest self-corrosion potential (Ecorr), suggesting more prone to corrode than other samples, and the largest corrosion current density of ZE21B alloy indicated that it possessed the fastest speed during the corrosion process. After the surface modification with MgF_2_, PDA, ECM 6 h, ECM 12 h, and ECM 24 h, all the samples showed higher corrosion potential and smaller corrosion current density compared with the bare ZE21B alloy, indicating better corrosion resistance function.

### 2.3. Hemocompatibility

**Figure 8:**
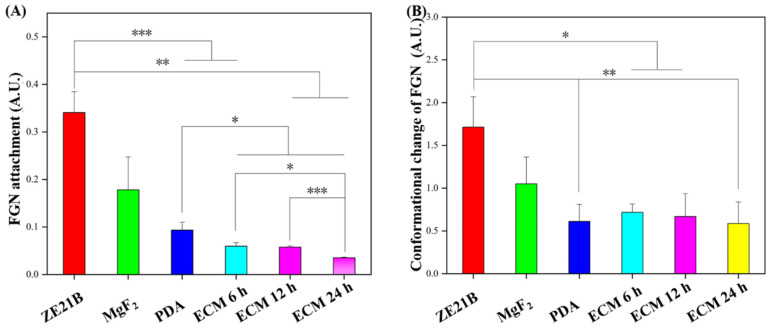
(**A**) Fibrinogen adhesion on ZE21B, MgF_2_, PDA, ECM 6 h, ECM 12 h, and ECM 24 h samples (mean ± SD, *n* = 5, * *p* < 0.05, ** *p* < 0.01, and *** *p* < 0.001); (**B**) Fibrinogen denaturation on ZE21B, MgF_2_, PDA, and different ECM samples (mean ± SD, *n* = 5, * *p* < 0.05, and ** *p* < 0.01).

After the implants contact the blood flow, fibrinogen (FGN) will be deposited on materials’ surfaces and cause platelets to adhesion and activation, leading to thrombus. Therefore, a typical ELISA kit method was used to determine adhesion and denaturation of FGN on the samples’ surfaces (Figure 8). The results showed that the absorbance values of adhesion and denaturation of the ECM group samples were significantly lower than those of the ZE21B samples. This result proved that the ECM samples possessed better function on inhibiting FGN adhesion and denaturation.

**Figure 9:**
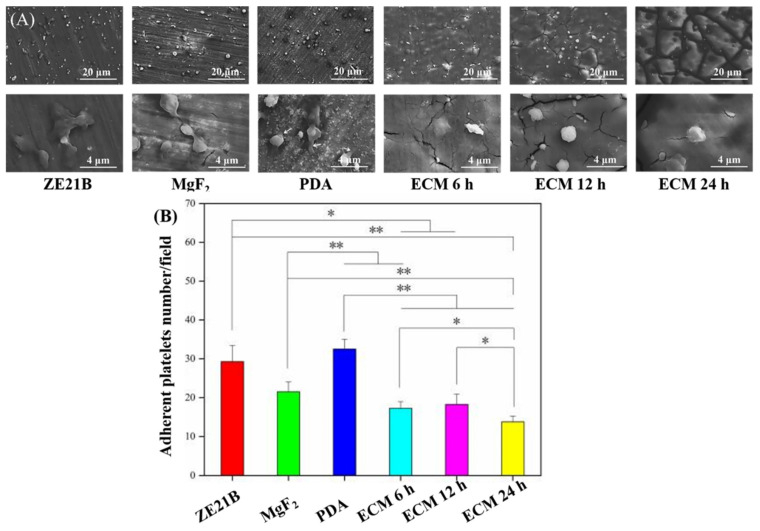
(**A**) SEM images of adherent platelets adhesion on ZE21B, MgF_2_, PDA, ECM 6 h, ECM 12 h, and ECM 24 h samples; (**B**) The number of adherent platelets on ZE21B, MgF_2_, PDA, and different ECM samples (mean ± SD, *n* = 8, * *p* < 0.05, and ** *p* < 0.01).

Excessive adhesion and activation of platelets can cause thrombosis, and here the platelets tests were performed (Figure 9). The SEM images (Figure 9A) showed that part of the platelets on the surfaces of the ZE21B and MgF_2_ samples appeared pseudopod formation, suggesting an activated state, while almost all the platelets on the surface of the PDA sample protruded their pseudopodia (marked by white arrow), which indicated that their anticoagulant function was insufficient. Only a small part of the platelets on the ECM 6 h samples have a spreading state, and the activated degree was lower than that of the PDA sample. However, most of the platelets on the ECM 12 h and ECM 24 h samples were spherical, and there was no obvious pseudopod, indicating that the latter two groups of samples had better function on inhibiting platelets activation. Quantitative characterization of platelets showed a trend of PDA > ZE21B > MgF_2_ > ECM 12 h and ECM 6 h > ECM 24 h, which indicated that the ECM group samples inhibited platelets adhesion on the Mg alloy. It has been reported that the different stages of cell adhesion, including attachment (and eventual detachment), flattening, and spreading, may be affected by series of physicochemical properties of the surface [24], such as the wettability, while the results of Figure 4 and Figure 9 showed that the surface wettability was not the only and absolute property affecting platelet adhesion. Generally speaking, hydrophilic surface is more conducive to platelet adhesion, but super hydrophilicity (WCA < 10°) often leads to platelet rupture and separation from the surface, reducing the number of platelets on the surface. In addition, the functional groups and charges on the surface will also affect the adhesion of platelets.

**Figure 10:**
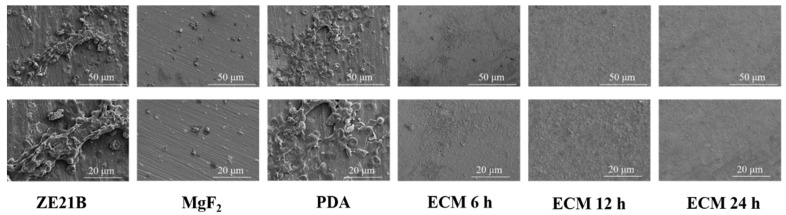
SEM images of components of blood adherent on ZE21B, MgF_2_, PDA, ECM 6 h, ECM 12 h, and ECM 24 h samples.

The SEM images of the whole blood components adherent on the samples showed that obvious thrombosis appeared on the ZE21B sample, suggesting poor anticoagulant ability. The MgF_2_ surface seemed clearer than ZE21B, but there were scattered platelets, all of which protruded pseudo foot, indicating the activated phenotype. Numerous platelets, red blood cells, and white blood cells adhered to the PDA surface, all of which may participate thrombogenesis. By comparison The ECM 6 h, ECM 12 h, and ECM 24 h samples presented clearest surfaces, which indicated the ECM coatings could improve the anticoagulant function of ZE21B alloy (Figure 10).

**Figure 11:**
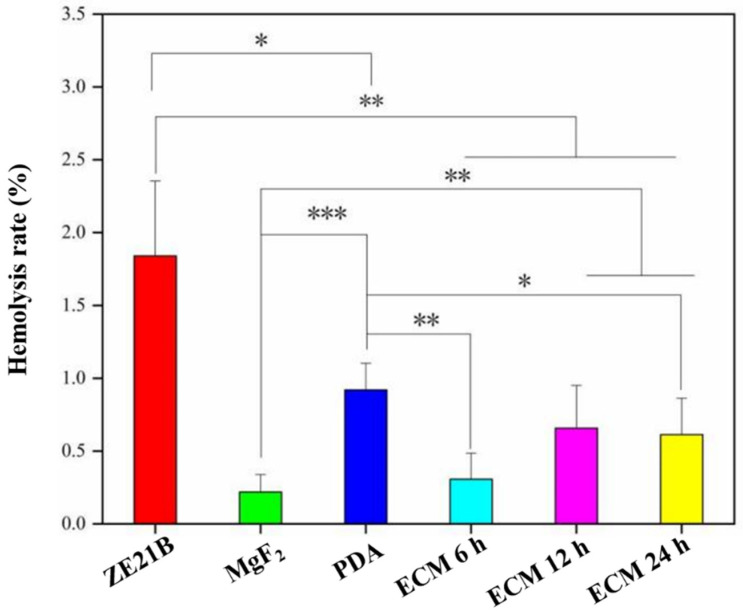
Hemolysis rates of ZE21B, MgF_2_, PDA, ECM 6 h, ECM 12 h, and ECM 24 h samples (mean ± SD, *n* = 5, * *p* < 0.05, ** *p* < 0.01, and *** *p* < 0.001).

Cardiovascular materials implant in the human body contact with blood that will cause the adhesion of red blood cells, and deformation and rupture of red blood cells may lead to hemolysis. Excessive hemolysis can cause thrombus and eventually block blood vessels. Therefore, hemolysis rate analysis of the different materials was tested here, and the results showed that all the samples exhibited their hemolysis rates less than 5%, which is a standard value for the blood contact materials, indicating no hemolysis. It has been reported that hydrophilic surfaces are more likely to lead to cell adhesion and cell membrane rupture [25], and this may be the reason ZE21B showed higher hemolysis rate than the other samples (Figure 11). However, the corrosion resistance and acid-base of the materials will affect the adhesion of cells and the rupture of cell membrane, which was why MgF_2_ sample showed lower hemolysis rate.

### 2.4. Pro-Endothelialization of the Nature-Inspired ECM Coating

**Figure 12:**
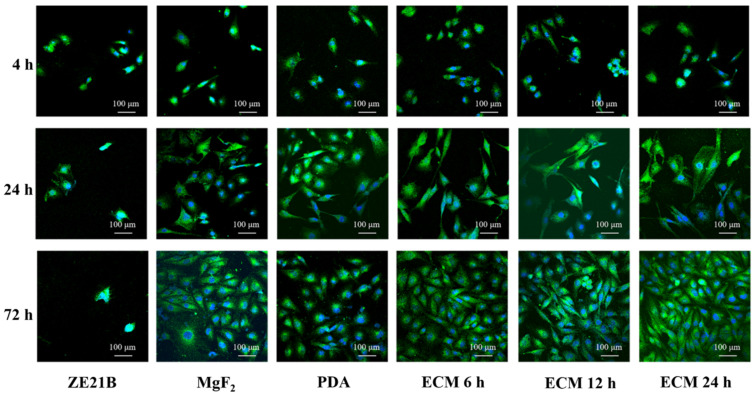
Fluorescence images of HUVECs in the ZE21B, MgF_2_, PDA, ECM 6 h, ECM 12 h, and ECM 24 h samples (The cells were stained with green color, and the nucleus were stained with blue color).

**Figure 13:**
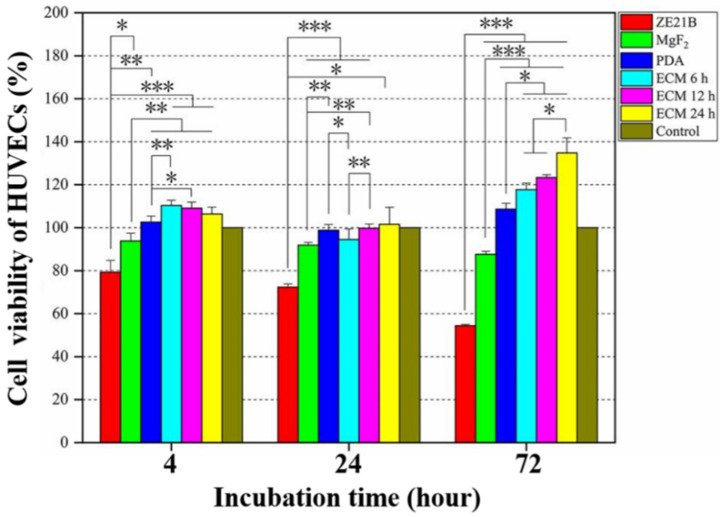
Cell viabilities of HUVECs in the ZE21B, MgF_2_, PDA, and different ECM groups at 4 h, 24 h, and 72 h (mean ± SD, *n* = 10, * *p* < 0.05, ** *p* < 0.01, and *** *p* < 0.001). The control group was RPMI-1640 complete medium (containing 10% serum and 1% penicillin and streptomycin mixture).

FN and Col IV provide ECs with cell membrane receptor ligands [26], which contribute to the rapid adhesion and proliferation of EC. Figure 12 showed that most of HUVECs showed a shrunken or round shape within the first 4 h, and all the modified layers could enhance the HUVECs number of ZE21B alloy. After 24 h, most of the HUVECs on the ZE21B alloy still showed a round shape, and the number of HUVECs reduced significantly, while the HUVECs of other groups not only increased in number, but also spread to different degrees. Some HUVECs presented fusiform morphology, indicating better activity, such as NO release [27,28]. After 72 h of incubation, the number of HUVECs on the ZE21B alloy decreased again, which may have been due to the excessive concentration of degradation products and inappropriate particle size from the pure ZE21B alloy [29]. Compared to the other samples, the ECM samples showed higher number and the larger spreading area of HUVECs, and the HUVECs exhibited elongated fusiform morphology. Figure 13 showed consistent results to the fluorescence images in Figure 12. All the coatings groups possessed higher HUVECs viability compared to ZE21B at the 4th hour and the 24th hour, and the ECM 24 h coating group presented higher HUVECs viability compared to the other groups.

**Figure 14:**
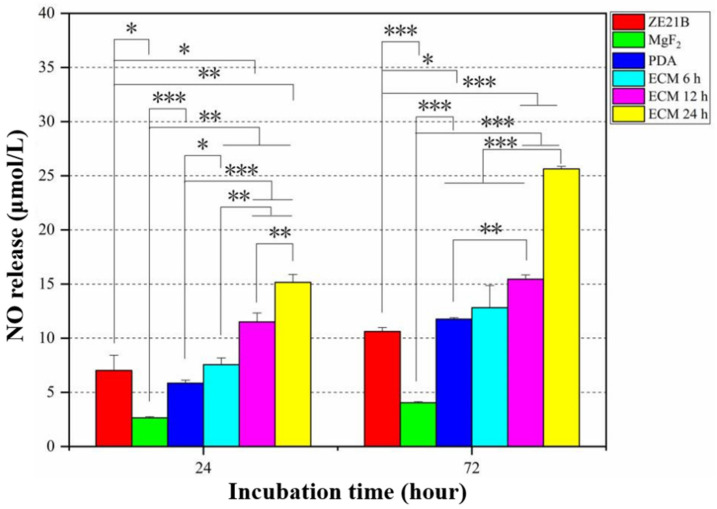
NO released by HUVECs in the ZE21B, MgF_2_, PDA, and different ECM groups (mean ± SD, *n* = 3, * *p* < 0.05, ** *p* < 0.01, and *** *p* < 0.001).

Nitric oxide (NO) is an important cell signaling molecule released by healthy physiological HUVECs [30]. It can regulate platelet aggregation [31], SMCs proliferation [32], and vasodilation [33]. Figure 14 shows the statistics of NO released by HUVECs in different groups for 24 h and 72 h. The HUVECs in the ECM groups released more NO compared to the HUVECs in the other groups, wherein the HUVECs in the ECM 24 h group released the largest amount of NO.

**Figure 15:**
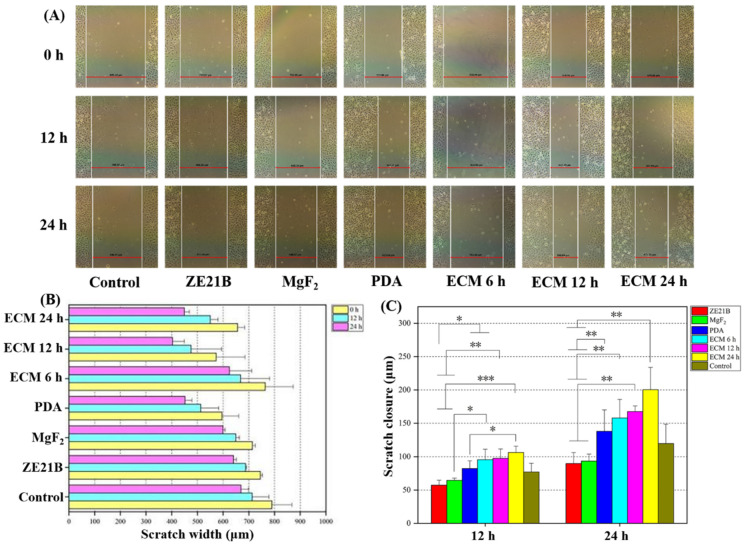
Migration experiment of HUVECs: (**A**) Optical microscope images at 0 h, 12 h, and 24 h; (**B**) Scratch width statistics result of each sample at 0 h, 12 h, and 24 h (mean ± SD, *n* = 5); (**C**) Total healing distance statistics result of each sample at 12 h and 24 h (mean ± SD, *n* = 5, * *p* < 0.05, ** *p* < 0.01, and *** *p* < 0.001). The control group was RPMI-1640 complete medium (containing 10% serum and 1% penicillin and streptomycin mixture).

EC migration is an important pathway for surface endothelialization of cardiovascular stents [34]. Here, a typical scratch experiment was performed to explore the migration ability of HUVECs on different samples. Figure 15 showed that the HUVECs in the ECM coatings group owned stronger migration ability compared with the HUVECs in the ZE21B group (Figure 15A,B). HUVECs in the ECM 24 h group exhibited stronger migration ability than the HUVECs in the other ECM coatings groups after 24 h (Figure 15C).

The HUVECs tests on proliferation, viability, NO release, and migration suggested the stronger pro-endothelialization function of ECM 24 h coating on the ZE21B.

### 2.5. Anti-Hyperplasia Function of the Nature-Inspired ECM Coating

**Figure 16:**
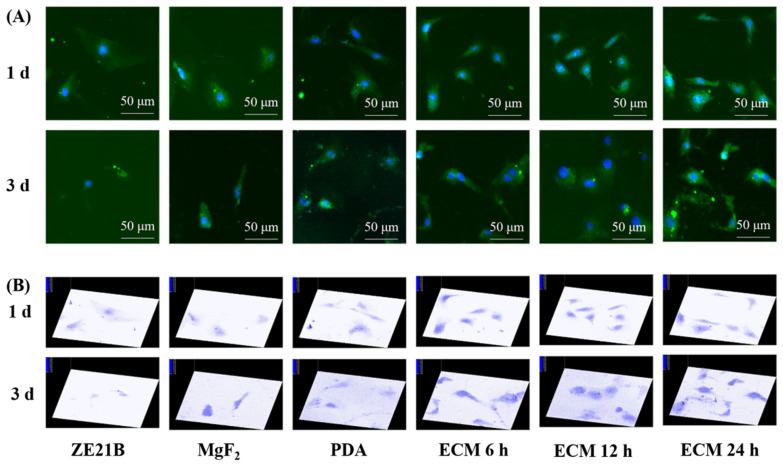
(**A**) Fluorescence images of SMC stained with α-SMA antibody (green) and DAPI (blue) on each sample; (**B**) Fluorescence intensity 3D images of α-SMA expression of SMC generated by Ipwin32 software.

SMC can be divided into contractile phenotype and synthetic phenotype [35]. Contractile SMCs were essential for the good performance of the vascular system and responsible for the normal physiological functions of arteries [35]. Synthetic SMC participate in vascular pathological process, thereby causing internal membrane hyperplasia and restenosis [35]. COL IV in ECM can promote the transition of SMC from a synthetic phenotype to a contractile phenotype [36]. The COL IV content on the ECM samples was different (Figure 6B), which may lead to a different regulation effect on the SMC phenotype change. Figure 16 showed that SMC in the ECM groups expressed stronger α-SMA (specific marker for contractile SMC) than SMC in the other groups, suggesting better anti-hyperplasia function [14].

### 2.6. Co-Culture of HUVECs and SMC

**Figure 17:**
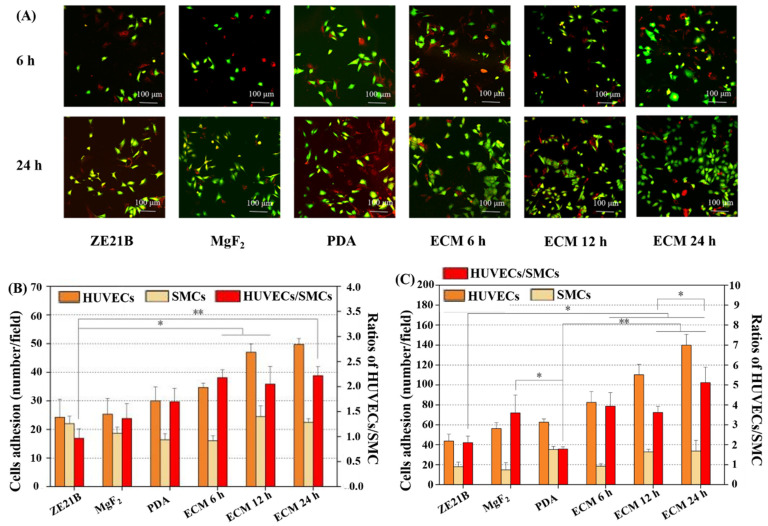
(**A**) CMFDA (green) staining of HUVECs and CFDA (red) staining of SMC on the surface of ZE21B, MgF_2_, PDA, and ECM samples; (**B**) After 6 h, the number of HUVECs and SMC on each sample, and the ratio of HUVECs/SMC (mean ± SD, *n* = 8, * *p* < 0.05, and ** *p* < 0.01); (**C**) After 24 h, the number of HUVECs and SMC on each sample and the ratio of HUVECs/SMC (mean ± SD, *n* = 8, * *p* < 0.05, and ** *p* < 0.01).

The growth of EC is affected by the complex vascular microenvironment, and the pericyte environment is an important factor to affect HUVECs [37]. SMC are important pericytes for EC [37]. Therefore, a co-culture model of HUVECs and SMC was built to evaluate their ability to compete for proliferation. As shown in Figure 17, HUVECs and SMCs are marked with green and red, respectively. After 6 h of co-culture, the ratios of HUVECs/SMC in the ECM 12 h group and ECM 24 h group were higher compared to the other groups. After 24 h of co-culture, the ECM 24 h showed the largest difference in the numbers of HUVECs and SMC, which made the ratio of HUVECs/SMC the highest, indicating that HUVECs in the ECM 24 h group had the strongest competitive growth advantage over SMC. Previous studies have shown that FN in the ECM can promote EC adhesion and proliferation [38]. The rapid proliferation of HUVECs may be related to it; COL IV in the ECM can inhibit the proliferation of SMC [39], which may endow the ECM coating strong anti-hyperplasia function. The reason why the ECM 24 h sample has the best endothelialization and anti-hyperplasia ability may be that it has more FN and COL IV (results in Figure 6).

### 2.7. Phenotype Change of Macrophages

**Figure 18:**
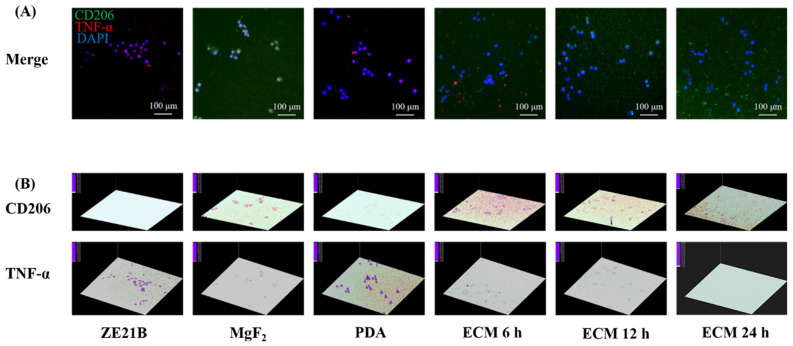
(**A**) Fluorescence images of macrophages stained with CD206 antibody (green), TNF-α antibody (red) and DAPI (blue) on each sample; (**B**) Fluorescence intensity 3D images of CD206 and TNF-α generated by Ipwin32 software.

Macrophages are an important immune cell in the human body. They are divided into two phenotypes: M1 and M2. Wherein, M1 phenotype participate in inflammatory response, and M2 phenotype play a function of resisting inflammation and promoting tissue regeneration physiologically [40]. Figure 18 showed that the macrophages in the ECM coatings groups expressed higher CD 206 and lower TNF-α compared with the macrophages in the other groups, suggesting better anti-inflammation function of the ECM coatings on the Mg alloy. The previous research has demonstrated that the M2 macrophages contributes to surface endothelialization [40]. Thus, the ECM coatings can also promote surface endothelialization of the Mg alloy via this pathway.

## 3. Materials and Methods

### 3.1. Surface Characterization

SEM (FEI Quanta 200, Eindhoven, Holland) and atomic force microscope (AFM, Bruker Dimension ICON, Berlin, Germany) were used to characterize the surface morphology and roughness of ZE21B, MgF_2_, PDA, as well as ECM 6 h, ECM 12 h, and ECM 24 h samples [41,42]. Fourier infrared spectroscopy (FTIR, Thermo Nicolet 6700, Thermo Fisher Scientific, Waltham, MA, USA) was used to analyze the functional groups on the sample surface [43]. Water contact angle measuring instrument (JC2000C, Powereach, Shanghai, China) was used to detect the water contact angle (WCA) of each sample to assess their hydrophilicity [44]. In order to evaluate the distribution of ECM on the samples, two major proteins in the ECM, type IV collagen (COL IV), and fibronectin (FN) [45,46] were selected to be characterized quantitatively and qualitatively.

### 3.2. Electrochemical Test

The electrochemical polarization curves of different samples were measured with RST 5200F electrochemical workstation. The start potential was set to −0.3 V, the end potential was set to 0.4 V, the scan rate was 0.001 V/s, and the voltage range to 2.0 V. Before the test, it is necessary to measure the open circuit voltage of the sample for 20 min, and then the Tafel curve could be measured. The three-electrode mode was used in the experiment: the auxiliary electrode, reference electrode, and working electrode are 260 platinum electrode, saturated calomel electrode, and the samples to be tested, respectively.

### 3.3. Hemocompatibility Test

For the fibrinogen adhesion and denaturation test, the blood of healthy volunteers was centrifuged at 3000 rpm for 15 min to obtain platelet-poor plasma superfluous (PPP). A total of 50 μL PPP was added to the surface of each sample and incubated at 37 °C for 1 h. Next, the samples were washed gently three times with NS, and 200 μL 1% BSA (Sigma, Toronto, ON, Canada) solution was added to block for 30 min. Next, 30 μL HRP-conjugated goat anti-human fibrinogen adsorbed/denatured antibodies (dilute antibody 500 times with PBS) was dropped on the surface of samples and incubated at 37 °C for 1 h. Thereafter, the samples were rinsed gently, and 100 μL TMB was added to react with the surface for 10 min in the dark; 50 μL of the 1 M sulfuric acid was added to stop the reaction. Finally, 120 µL of the solution on the sample surface was transferred to a 96-well plate to measure the absorbance at 450 nm with a microplate reader [47].

The blood of healthy volunteers was centrifuged at 1500 rpm for 15 min to obtain platelet-rich plasma supernatant (PRP). A total of 80 μL PRP was added to the surface of each sample and incubated at 37 °C for 1 h. Then, the samples of each group were gently washed three times with NS, and 1 mL 4% paraformaldehyde solution was added to fix the platelets at room temperature (RT) for 30 min. After the washing step, the samples were dehydrated gradually using 25%, 50%, 75%, and 100% ethanol solution. During a complete drying step and spraying gold treatment, the platelets adherent on the samples were observed by SEM; the numbers of the platelets adherent on each sample were counted according to the previous report [48].

To investigate the overall anticoagulant function of each sample, a whole blood experiment was performed. An amount of 600 µL of fresh healthy adult blood containing 67 µL of sodium citrate solution was added to each sample and incubated in a shaker at 37 °C for 1 h. Next, the samples were slowly rinsed for 3 times with PBS, and 4% paraformaldehyde solution was added to fix the sample for 30 min. After the washing step, the samples were dehydrated by gradient dehydration with different concentrations of alcohol and followed with drying. Finally, the samples were sprayed with gold and observed by SEM [43].

Fresh human blood and NS in a ratio of 4:5 was diluted for later hemolysis assessment. Firstly, the samples were treated with silica gel on the side and bottom, and then they were placed in 10 mL NS. At the same time, 10 mL NS group and ultrapure water group without samples were set as the positive and negative control group of the experiment. They were then placed in a constant temperature water bath at 37 °C and incubated for 30 min. Next, 0.2 mL of diluted blood was added to the experimental group and the control group, respectively, and incubated for 1 h. The samples were taken out and the remaining solution was centrifuged at 2500 rpm for 5 min. Finally, 150 μL of the supernatant was transferred to a 96-well plate to measure the absorbance value at 540 nm with a microplate reader, and the hemolysis rate of each sample was calculated according to Equation (1) [19].

Equation: Hemolysis rate = [(ODt − ODn)/(ODp − ODn)] × 100%(1)

where ODt is the absorbance value of the test group; ODn is the absorbance value of the negative control group; ODp is the absorbance value of the positive control group.

### 3.4. Culture and Evaluation of EC

Extracts of each samples were prepared in accordance with the requirements of ISO 10993-12: the sides and back of the samples were sealed with silica gel. The front and back sides of the samples were irradiated using ultraviolet light for 30 min. The samples were then put into a 24-non-adhesive well plate, and immersed in RPMI-1640 medium without fetal bovine serum and penicillin followed by the ratio of 1.25 cm^2^/mL (628 µL) and placed in a constant temperature incubator at 37 °C and 5% CO_2_. After 24 h, the extracts of each group were collected for later use.

Human umbilical vein endothelial cells (HUVECs, purchased from Haoyi Biotechnology Co., Ltd., Chengdu, China) were seeded on a cell slide with a diameter of 8 mm at a concentration of 1.5 × 10^4^ cells/mL, and then 1 mL complete medium (containing 10% serum and 1% penicillin and streptomycin mixture) was added to culture HUVECs for one day. After removing the used medium, 500 µL fresh RPMI-1640 complete medium, 500 µL extracts, 50 µL fetal bovine serum, and 5 µL penicillin were added at the same time. The above operation was repeated every day. At the 4 h, 24 h, and 72 h after cultured using the extracts, the HUVECs were fixed with 4% paraformaldehyde (Sigma) at RT and stained with rhodamine and DAPI, and observed under a confocal laser scanning microscope (CLSM, Nikon C2 Plus, Tokyo, Japan) [49]. The viability and nitric oxide (NO) release of HUVECs were detected by a typical ELISA method and calculated according to the previous study [50,51].

The physical scratch method is used to study the migration of HUVECs in different groups. Firstly, the HUVECs were seeded in a 24-adhesive well plate at a concentration of 3 × 10^4^ cells/mL. When the cell grows to about 90%, a 10 µL pipette tip was used to draw a line on the bottom of the well plate. Next, the extracts of different samples were added into the plate, and the changes about the width of the scratches at the time points of 0 h, 12 h, and 24 h to evaluate the migration of HUVECs in each group was recorded.

### 3.5. Cultivation and Evaluation of SMC

In this study, the SMC test was performed using the extracting solution of each sample. Here, DMEM high-sugar medium (icell) is used instead of RPMI-1640 medium in Section 2.4, and other operations are the same as HUVECs culture. After the SMC were cultured for 4 h, 24 h, and 72 h the BSA blocking solution was added and incubated at RM for 30 min, and then 0.5% Triton X-100 solution was added to permeate for 5 min. Afterwards, 120 µL FITC-labeled phalloidin working solution (200 nmol/L) was added and incubated for 30 min in the dark. After rinsing the sample with NS, 80 µL DAPI solution added to stain the nucleus in the dark for 4 min. Finally, the morphology of SMC was observed by CLSM. The viability of SMCs was also detected and calculated.

The phenotypic changes of SMC not only determine its pathological/physiological properties, but further affect endothelialization [52,53]. In this experiment, after cultured for 24 h and 72 h, respectively, the SMC were stained with α-smooth muscle actin (α-SMA, marker for contractile SMC) and DAPI to assess their phenotypic changes [54]. After recording the immunofluorescence images with a fluorescent inverted microscope (Ti-S, Nikon, Tokyo, Japan), the 3D images of fluorescence intensity generated by Ipwin32 software as a semi-quantitative characterization were obtained [55].

### 3.6. Co-Culture of HUVECs and SMC

A biocompatible coating needs to simultaneously promote HUVECs growth and inhibit the excessive proliferation of SMC [56]. Therefore, in order to more intuitively observe the interaction of HUVECs and SMC on the ECM coated ZE21B alloy, a co-culture experiment was performed. In brief, HUVECs and SMC were labeled with two tracers in advance (HUVECs were labeled with CytoTraceTM Green CMFDA, and SMC were labeled with CytoTraceTM Red CFDA). The densities of both cells were adjusted to 1 × 10^4^ cells/mL with DMEM complete medium, and then the HUVECs and SMC were mixed at a ratio of 1:1 and seeded on the cell slide, subsequently cultured with the extracts solution. After cultured for 6 h and 24 h, respectively, the behaviors of the co-cultured HUVECs and SMC were observed by CLSM.

### 3.7. Culture and Evaluation of Macrophages

The macrophages (purchased from Haoyi Biotechnology Co., Ltd., Chengdu, China) were seeded on each sample surface at a density of 1 × 10^4^ cells/mL and cultured at a standard condition for one day [57]. After stained with TNF-α antibody (marker for the M1 macrophages), CD206 antibody (marker for the M2 macrophages), and DAPI, the cell number and phenotypic changes were recorded by CLSM, and the 3D images of the fluorescence intensity of TNF-α and CD206 of macrophages on different samples were generated by Ipwin32 software as semi-quantitative characterization [58].

## 4. Conclusions

In this contribution, the ECM that biomimetic vascular basement membrane was successfully synthesized and covalently attached to the biodegradable Mg alloy (ZE21B) surface as a multi-functional coating. The platelets/FGN tests and hemolysis experiment indicated that the nature-inspired ECM coating could significantly improve the blood compatibility of the Mg alloy. The cytocompatibility result demonstrated that this composite ECM endowed the Mg alloy better pro-endothelialization, anti-hyperplasia, and anti-inflammation functions. Notably, this study overcome the application limitation of the ECM secreted by HA micro-patterned SMC and EC, which is not suitable for direct preparation on the surface of degradable materials and presented the self-assembled ECM as a feasible and promising strategy for the surface modification of the biodegradable Mg alloy stent for cardiovascular stent.

## Figures and Tables

**Table 1:** The corrosion potential (Ecorr) and current density (Icorr) values of ZE21B, MgF_2_, PDA, ECM 6 h, ECM 12 h, and ECM 24 h samples.

Samples	Ecoor (V)	Icoor (A/cm^2^)
ZE21B	−1.535	2.529 × 10^−6^
MgF_2_	−1.383	2.209 × 10^−7^
PDA	−1.457	1.461 × 10^−7^
ECM 6 h	−1.500	3.722 × 10^−7^
ECM 12 h	−1.440	4.240 × 10^−7^
ECM 24 h	−1.474	6.307 × 10^−7^

## Data Availability

The data presented in this study are available on request from the corresponding author.
